# Editorial: The interplay between food and intestinal microbiota: How they impact on the well-being status of the host

**DOI:** 10.3389/fmicb.2022.980243

**Published:** 2022-07-15

**Authors:** Marina Liso, Rachele De Giuseppe, Erica Pontonio

**Affiliations:** ^1^National Institute of Gastroenterology “S. de Bellis” Research Hospital (IRCCS), Bari, Italy; ^2^Laboratory of Dietetics and Clinical Nutrition, Department of Public Health, Experimental and Forensic Medicine, University of Pavia, Pavia, Italy; ^3^Department of Soil, Plant, and Food Sciences, University of Bari Aldo Moro, Bari, Italy

**Keywords:** food, inflammation, microbiota, nutrients, immunity, diet

The Research Topic aimed to present a novel approach and findings explaining the role of different food components in shaping the gut microbiota composition and intestinal homeostasis, to understand the relationship between microbial metabolites with immunity and epithelial integrity.

The study of gut microbial modifications in association with changes of diet administration has made it possible to identify a deep relationship between these two factors and the wellbeing status of the host.

## Effect of natural compounds on immunity and microbiota composition

The intestinal tract of a healthy body is home to a large variety and number of microorganisms that will affect every aspect of the host's life, and it can be affected by several factors. Indeed, the role of naturally derived compounds in regulating the intestinal microbiota composition as well as their effect on the homeostasis, chronic inflammatory conditions, and the immune response has recently been investigated.

Among the natural compounds, polysaccharides have been found to be an important factor affecting intestinal microbiota. In the intestinal tract of living organisms, polysaccharides have many important functions, such as maintaining normal intestinal microbiota structure, as well as improving cognitive function in the brain *via* the brain-gut axis by virtue of the intestinal microbiota. Also, polysaccharides may play a significant role in the treatment of the pathogenesis of depression (Wang X. et al.).

The study of Geng et al. shows the effect of dietary modified Bazhen powder (MBP), a traditional Chinese herbal prescription, on immunity and reproductive performance in sows. They administered MBP to pure-bred Yorkshire sows, during gestation and lactation. The MBP supplementation promotes immunoglobulin synthesis and improved gastrointestinal function, by increasing the serum level of gastrin and motilin, important for nutrient digestion and absorption (Walsh, 1990; Al-Missri and Jialal, 2021). MBP supplementation affects the metabolic and microbial content of milk, and increases its α-diversity. Albiflorin and ursolic acid, with immunoregulatory and anti-inflammatory activities (Fei et al., 2016; Wang et al., 2018), were higher in the MBP group. This study demonstrated that MPB should be considered a potential nutritional supplementation during gestation and lactation for perinatal mothers.

The cardiovascular protective effect of the natural compound Berberine (BBR), a plant alkaloid extracted from *Berberis vulgaris* and *Coptis chinensis*, was investigated by Wang Z. et al. Angiotensin II-induced hypertensive mice were treated with BBR or choline. BBR decreased blood pressure and improved vascular function of the abdominal aorta in Ang II-induced hypertensive mice. The gut microbiota of mice was also affected by BBR. TMAO (trimethylamine-N-oxide), a bioactive metabolite produced by gut microbiota, has been previously associated with cardiovascular diseases and hypertension risk (Ge et al., 2020). A reduction of plasma TMAO was recorded in the BBR treated group. TMAO was able to promote apoptosis and oxidative stress *in vitro*. This study demonstrated that BBR exerts its protective role in hypertension *via* modulation of gut microbial composition and inhibition of TMAO synthesis.

## Anti-inflammatory properties of bacterial metabolites

*Limosilactobacillus fermentum* (previously known as *Lactobacillus fermentum*) is a probiotic bacterium producing antioxidants and short-chain fatty acids, with a direct effect on regulating the intestinal homeostasis and immune response (Peran et al., 2006; Sun et al., 2017; Jang et al., 2019). Su et al. explored the *in vitro* and *in vivo* anti-inflammatory activity of metabolites of *Lactobacillus fermentum* F-B9-1 (MLF), isolated from the soil. MLF treatment on Caco-2 cells reduced the level of pro-inflammatory cytokines in the cellular supernatant and improved the tight junction integrity. In a mouse model of DSS-induced colitis, MLF administration confirmed the effects on pro-inflammatory cytokines and tight junction shown *in vitro*. The clinical symptoms of colitis were also reduced in the MLF group, as well as the gut microbiota dysbiosis. This study paves the way for using MLF as a potential probiotic supplement in colitis-affected patients.

## The role of gut mycobiome in host metabolic homeostasis

Several studies in the last decades have demonstrated the role of gut microbiota in health and disease, and its contribution to the maintenance of homeostasis in metabolic disorders such as obesity and type 2 diabetes (Crusell et al., 2018; Wu et al., 2021; Bielka et al., 2022). However, the contribution of gut mycobiome has been less investigated. Wu et al. characterized the gut mycobiome profile in gestational diabetes mellitus (GDM) and healthy subjects in the second trimester of pregnancy, demonstrating a reduction of β-diversity in the GDM group. Specific fungal taxa were more abundant in the GDM group, demonstrating an association with abnormal blood glucose metabolism. GDM patients received diet intervention for 2 weeks which resulted in an increase of the probiotic genus *Ganoderma*, with anti-inflammatory properties previously demonstrated in mice (Lee et al., 2020; Shao et al., 2022; Wen et al., 2022). This study points out the role of gut mycobiome in metabolic disorders and provides a new alternative strategy for GDM patient's management, through a short-term diet intervention.

## Metabolomics approach to celiac disease

Celiac disease (CD) is an inflammatory and immune-mediated enteropathic disorder that primarily involves the small intestine of genetically predisposed individuals (Lebwohl et al., 2018; Catassi et al., 2022). CD is triggered by the ingestion of gluten found in wheat, rye and barley, which maintains small intestinal mucosal injury (Kagnoff, 2005; Fasano and Catassi, 2012; Elli et al., 2019). Thus, a gluten-free diet (GFD) seems to be an effective way of CD treatment and enables the majority of patients to achieve clinical and histological remission. It has been reported that intestinal dysbiosis might be involved in the CD pathogenesis; indeed, the impact of a GFD on the gut microbiome composition on CD patients has been described (Kaliciak et al., 2022).

Based on these considerations, Vacca et al. summarized in a critical narrative review the main metabolomic variations related to the interplay between host, gut microbiota and diet, suggesting the chance to exploit specific metabolites as biomarkers for a non-invasive diagnosis of CD. However, the authors reported that it is not possible to provide an unambiguous statement on a CD-related metabolomics footprint due to the great heterogeneity of the study designs, the methodological approaches applied and the limited number of research articles. Although there is a growing interest in exploring the triggers of CD through meta-omic approaches, the prospect of having a diagnosis of CD by applying a non-invasive metabolomics investigation is still a long way off considering the multiple forms of expression of gluten-related disorders (Vacca et al.).

## Author contributions

All authors listed have made a substantial, direct, and intellectual contribution to the work and approved it for publication.

## Conflict of interest

The authors declare that the research was conducted in the absence of any commercial or financial relationships that could be construed as a potential conflict of interest.

## Publisher's note

All claims expressed in this article are solely those of the authors and do not necessarily represent those of their affiliated organizations, or those of the publisher, the editors and the reviewers. Any product that may be evaluated in this article, or claim that may be made by its manufacturer, is not guaranteed or endorsed by the publisher.
